# An Atypical Case of Neuroleptic Malignant Syndrome Associated With Ciprofloxacin and Quetiapine

**DOI:** 10.7759/cureus.36178

**Published:** 2023-03-15

**Authors:** Muhammad U Rohail, Ayub Khan, Rhani M Pflaum, Mitul Patel, Melissa A Moody

**Affiliations:** 1 Medical School, West Virginia School of Osteopathic Medicine, Lewisburg, USA; 2 Psychiatry, Boone Memorial Health, Madison, USA

**Keywords:** serotonin syndrome (ss), quetiapine, ciprofloxacin, neuroleptic malignant syndrome (nms), second generation antipsychotics

## Abstract

A 29-year-old male presented to the emergency department with complaints of shortness of breath and numbness in bilateral upper and lower extremities that started a few hours prior to arrival. On physical examination, the patient was afebrile, disoriented, tachypneic, tachycardic, and hypertensive with generalized muscle rigidity. Further investigation revealed that the patient had recently been prescribed ciprofloxacin and restarted on quetiapine. The initial differential diagnosis was acute dystonia, and subsequently, the patient was placed on fluids, lorazepam, diazepam, and later benztropine. The patient’s symptoms began to resolve, and psychiatry was consulted. Given the patient’s autonomic instability, altered mental status, muscle rigidity, and leukocytosis, psychiatric consultation revealed an atypical case of neuroleptic malignant syndrome (NMS). It was postulated that the patient’s NMS was caused by a drug-drug interaction (DDI) between ciprofloxacin, a moderate cytochrome P450 (CYP) 3A4 inhibitor, and quetiapine, which is primarily metabolized by CYP3A4. The patient was then taken off quetiapine, admitted overnight, and discharged the next morning with complete resolution of his symptoms along with a prescription for diazepam. This case highlights the variable presentation of NMS and the need for clinicians to consider DDI when managing psychiatric patients.

## Introduction

Neuroleptic malignant syndrome (NMS) is a rare and life-threatening form of dysautonomia caused by a reduction in dopaminergic activity in central nervous system (CNS) pathways. Primary causes of NMS include excess dopamine receptor antagonism from antipsychotics and abrupt withdrawal of dopamine activity from Parkinson’s disease medications and other dopamine receptor agonists. Few cases reporting drug-drug interaction (DDI) between a second-generation antipsychotic (SGA) and antibiotics exist in the literature [1-3]. Here, we present the case of a patient who was prescribed ciprofloxacin for a urinary tract infection (UTI) shortly after being restarted on quetiapine and subsequently developed NMS.

## Case presentation

A 29-year-old male with a history of intellectual disability, recurrent nephrolithiasis, asthma, bipolar-like symptoms, and generalized anxiety disorder presented to the emergency department with a chief complaint of shortness of breath that began a few hours prior to presentation. Shortly after arrival, the patient reported numbness in bilateral upper and lower extremities and some mild chest pain. He denied any other symptoms. He also denied any recent substance use. On physical examination, the patient was afebrile, hypertensive (136/98 mmHg), tachycardic (114 beats/minute), and tachypneic (26 respirations/minute). He was also disoriented with generalized muscle rigidity. His arterial blood gases demonstrated respiratory alkalosis and compensatory metabolic acidosis. Lab work revealed elevated lactate and leukocytosis. Creatine kinase (CK), troponin, and electrocardiogram (EKG) were unremarkable (Table 1). After an initial examination, the patient was given fluids, followed by lorazepam and diazepam. His symptoms transiently resolved. Upon chart review, it was noted that two days earlier, the patient had been empirically prescribed ciprofloxacin and ondansetron for a UTI by his primary care provider. The patient was also recently restarted on quetiapine, bupropion, and buspirone by his psychiatrist. Additional doses of lorazepam and diazepam were administered alongside benztropine.

**Table 1:** Patient laboratory values suggestive of neuroleptic malignant syndrome. PCO_2_: partial pressure of carbon dioxide; HCO_3_: bicarbonate.

Laboratory values	Results	Reference values
pH	7.74	7.35–7.45
PCO_2_	12	35–48 mmHg
HCO_3_	16	21–29 mmol/L
White blood count	11	4.8–10.8 × 10^3^/μL
Troponin I	<0.010	0.000–0.040 ng/mL
CK	165	49–269 IU/L
Lactate	3	0.5–2.2 mmol/L

Psychiatry was then consulted for the acute dystonia-like symptoms and the concern for possible NMS caused by a DDI. In discussion with psychiatry, the patient was determined to have a high likelihood of NMS given a Delphi score of 62 and his clinical presentation. NMS was believed to be caused by interactions between ciprofloxacin and quetiapine. Per psychiatry’s recommendations, the patient was taken off bupropion, quetiapine, and buspirone. The patient responded well to the treatment, with a resolution of symptoms and a return to stable vitals. He was later admitted for observation overnight and was subsequently discharged the next day with diazepam as needed.

The patient followed up a week later with his psychiatrist and did not report having any symptoms at that time. He was then restarted on bupropion for mood stabilization and smoking cessation.

## Discussion

NMS is a rare and potentially fatal iatrogenic dysautonomia, with an incidence rate of 0.02% to 3% for patients taking antipsychotic drugs. Age does not appear to be a risk factor; however, there is a 2:1 ratio of males to females with a diagnosis of NMS [2]. The most common causative agents are first-generation antipsychotics. SGAs, such as clozapine, have also been shown to cause NMS, although with a much lower incidence [1]. Our case presents an atypical presentation of NMS caused by quetiapine, an SGA.

Although the pathophysiology of NMS is rather unclear, it is thought to be caused by an abrupt reduction in dopaminergic activity. This may be due to a sudden increase in the antagonism of dopamine subtype 2 (D2) receptors from antipsychotics or a decrease in dopamine receptor agonist activity [1-3]. Literature suggests that the blockade of D2 receptors in the hypothalamic, nigrostriatal, and mesolimbic/cortical pathways causes the neuromuscular and neurological symptoms of NMS. It is also suggested that dopamine has a regulatory role in the sympathetic system and that the sudden decrease in dopaminergic activity contributes to the autonomic instability in the NMS [1].

NMS is a clinical diagnosis that presents as a constellation of symptoms that typically include fever, altered mental status, muscle rigidity, and autonomic instability. Blood work can also reveal leukocytosis and elevated CK values secondary to rhabdomyolysis. Autonomic instability in NMS presents a wide spectrum of symptoms, and this often makes diagnosis difficult [1,2]. Our patient did not present with a fever, and lab work did not reveal elevated CK values. Literature suggests that hyperthermia may be a later presenting and less consistent symptom present in patients experiencing NMS secondary to SGAs [2]. This may explain the absence of fever in our patient. He did, however, demonstrate muscle rigidity, an altered mental status, tachycardia, tachypnea, and elevated blood pressure. This was consistent with common presentations of NMS. NMS currently does not have universally recognized diagnostic criteria, but the Delphi method and Diagnostic and Statistical Manual of Mental Disorders, Fifth Edition (DSM-V) are often used to aid in clinical diagnosis [4,5]. Using the Delphi method, our patient’s recent exposure to a dopamine antagonist, muscle rigidity, altered mental status, hypermetabolism, and negative toxic, metabolic, and neurologic workup demonstrated a score of 62 out of 100 [4].

When treating NMS, it is imperative to continue monitoring and maintaining cardiorespiratory stability with concomitant symptomatic treatment. The initial treatment is the withdrawal of the offending agent. Management is then focused on the symptomatic treatment of NMS and may include cooling techniques for hyperthermia, fluid resuscitation for rhabdomyolysis, electrolyte management for acid/base abnormalities, and benzodiazepines to control muscle rigidity and agitation [1,2]. For our patient, quetiapine was withheld, and he was treated with lorazepam and diazepam. This was consistent with treatment guidelines. In addition, our patient was also treated with benztropine due to an early assessment of muscle rigidity as possible acute dystonia, and it is unclear if this was beneficial in treating our patient’s symptoms.

NMS is a difficult diagnosis to make due to symptoms that are consistent with several other dysautonomias. When evaluating the patient, it is important to think about and rule out other life-threatening possibilities. A thorough review of medication history is helpful in differentiating these different pathologies. Our initial differential was acute dystonia; however, due to the patient’s autonomic instability, leukocytosis, and metabolic acidosis, a more apt diagnosis of NMS was made. Serotonin syndrome is a common differential primarily caused by excess serotonergic activity secondary to selective serotonin reuptake inhibitors that can be differentiated from NMS with distinctive symptoms of hyperreflexia, shivering, ataxia, and myoclonus. Malignant catatonia is another differential that is caused by underlying psychiatric conditions and can be worsened by antipsychotic therapy; however, it is typically preceded by weeks of positive catatonic and behavioral symptoms [1,2]. These include waxy flexibility, dystonic posturing, and repetitive movements. Thyrotoxicosis is an additional differential that was ruled out due to an absence of a medical history of thyroid disease or thyroid medication and an unremarkable thyroid panel. Lastly, illicit drug intoxication, such as methamphetamine, cocaine, ecstasy, and phencyclidine, was all ruled out due to a negative drug screen [2].

In our case, the patient's recent prescription of ciprofloxacin for his UTI was considered to have interacted with quetiapine. Quetiapine is predominantly metabolized in the liver by hepatic cytochrome P450 (CYP) enzymes, namely CYP3A4 and CYP2D6. Ciprofloxacin is known to be a moderate CYP3A4 inhibitor, which the authors believe caused an increase in the plasma concentration of quetiapine, leading to an atypical presentation of NMS [6]. This subsequent supratherapeutic level of quetiapine in our patient may have led to excess D2 receptor antagonism in a short period of time, ultimately disrupting the aforementioned dopaminergic CNS pathways and causing the distinct neuromuscular and autonomic symptoms of NMS. Other potential interactions between the two include prolongation of the corrected QT interval (QTc), which ultimately can lead to arrhythmias such as torsades de pointes and death [7]. Our patient did not have a prolonged QTc interval on his EKG.

## Conclusions

NMS is a life-threatening dysautonomia that classically presents with autonomic and neuromuscular instability. It is a condition often precipitated by the supratherapeutic pharmacodynamics of antipsychotic medications. Various DDIs can alter the plasma concentration of these agents. Our case involved interactions between ciprofloxacin and quetiapine that the authors believe ultimately caused NMS in the patient. Therefore, it is critical for clinicians to assess for DDIs when managing patients receiving psychiatric treatment.
